# Supplementary material for: Tunable Picoliter‐Scale Dropicle Formation Using Amphiphilic Microparticles with Patterned Hydrophilic Patches
Source: Adv Sci (Weinh). 2024 Dec 24;12(12):2411014. doi: 10.1002/advs.202411014 (PMC11948056; doi:10.1002/advs.202411014)
Supplement: Supplementary file 1 — Supporting Information [file ADVS-12-2411014-s002.pdf]

ADVANCED SCIENCE
Open Access

Supporting Information

for *Adv. Sci.*, DOI 10.1002/advs.202411014

Tunable Picoliter-Scale Dropicle Formation Using Amphiphilic Microparticles with Patterned Hydrophilic Patches

Xinpei Song, Shreya Udani, Mengxing Ouyang, Mehmet Akif Sahin, Dino Di Carlo and Ghulam Destgeer**

Supporting information

Tunable Picoliter-scale Dropicle Formation using Amphiphilic Microparticles with Patterned Hydrophilic Patches

Xinpei Song[1+], Shreya Udani[2+], Mengxing Ouyang[2], Mehmet Akif Sahin[1], Dino Di Carlo[2*], and Ghulam Destgeer[1*]

[1]Control and Manipulation of Microscale Living Objects, Center for Translational Cancer Research (TranslaTUM), Munich Institute of Biomedical Engineering (MIBE), Department of Electrical Engineering, School of Computation, Information and Technology (CIT), Technical University of Munich, Einsteinstraße 25, Munich 81675, Germany.

[2]Department of Bioengineering, University of California Los Angeles, Los Angeles, CA 90095, USA.

[+]Equal contributions. *Corresponding authors: dicarlo@ucla.edu, ghulam.destgeer@tum.de

Numerical simulations to predict particle shape:

We obtained the flow streamlines at the outlet of the microfluidic device for predicting the F_1, F_2, F_4, F_9, F_{12}, and F_{16} particles' cross-section shapes using the single-phase (**Figure S1**A) and two-phase (Figure S1B) laminar flow modules in COMSOL Multiphysics. In the numerical results, the predicted shapes of the F_1, F_2, and F_4 particles had some differences, whereas the F_9, F_{12}, and F_{16} particle shapes were similar.

Apparently, the simulated particle shapes using the single-phase module were similar to the experimentally measured particles in EtOH, while the two-phase flow simulation results were closer to the particles in PBS. However, it should be noted that the predicted particle shape corresponds to the cross-sectional shape of the flow streams in the liquid state at the outlet of the microfluidic device. Once the precursor streams are cured under UV exposure, the solid particle shape will have slight differences from the predicted shape in the numerical results. Moreover, the cured particles were later washed with EtOH and transferred to PBS for experimental imaging. The media exchange also affected the particle shape. In conclusion, the two-phase flow simulation should have a more realistic prediction of the particle shape, as this model accommodates the variable viscosities of the co-flowing streams. However, the numerical and experimental results should be compared with caution and should not be expected to strictly match.

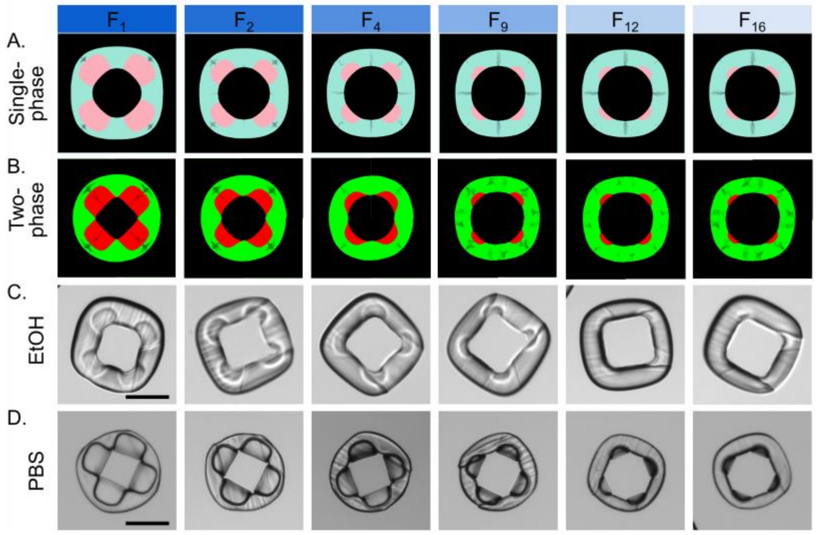

Figure S1. Simulated cross-section of the 4C particles using (A) single-phase and (B) two-phase flow modules. Experimentally observed particles in (C) EtOH and (D) PBS. Scale bar: 200μm.

Numerical simulations for dropicle formation within the 4C amphiphilic microparticle:

We integrated all the numerical results of the dropicle formation in **Figure S2**. Two different C^{4d} dropicles, split from the R^{+2d} dropicle, were numerically obtained for the same 4C particle, with large and small hydrophilic patches, at H_d/H_c = 0.28 and 0.25, respectively. The first type ($[C^{4d}]_I$) had four droplets attached to the hydrophilic patches at the corners, one significantly larger than the other three. For the second type ($[C^{4d}]_{II}$), four attached droplets had relatively similar volumes. The unattached central 5th droplet was regarded to be washed away by the oil in the experiment, eventually, resulting in four similar droplets attached at the corners. However, we didn't observe $[C^{4d}]_I$ dropicle within the 4C particle with medium hydrophilic patches, indicating that the transition from $[C^{4d}]_I$ to $[C^{4d}]_{II}$ was uncertain.

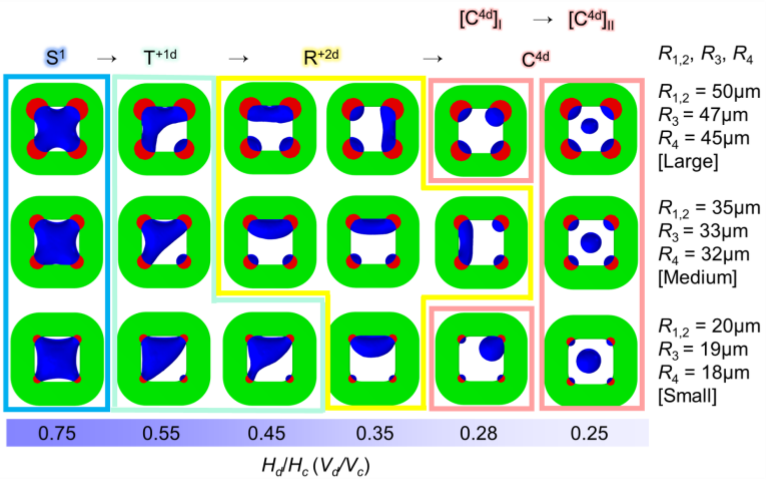

Figure S2. In the asymmetric model, the hydrophilic patch radii ($R_{1\text{-}4}$) are varied from large to medium to small combinations to realize the dropicle transition from $S^1 \rightarrow T^{+1d} \rightarrow R^{+2d} \rightarrow C^{4d}$ for variable H_d/H_c ratio. Two types of the C^{4d} dropicles are obtained, $[C^{4d}]_I$ and $[C^{4d}]_{II}$.

Numerical simulations for dropicle formation within amphiphilic microparticles of other shapes:

We have numerically investigated amphiphilic microparticles with triangular, square (as in the main manuscript), circular, pentagonal, and hexagonal shapes with three, four, four, five, and six hydrophilic patches, respectively, for their ability to form dropicles within their inner cavities (**Figure S**3). The diameters of the inscribed circles, tangent to the inner and outer boundaries of these particles, were 400 µm and 200 µm, respectively, which were consistent with the particle diameter D_p and cavity diameter D_c of the 4C amphiphilic microparticle in the main manuscript. The geometric centers of the hydrophilic patches were evenly patterned at the triangular, square, circular, pentagonal, and hexagonal outlines of these amphiphilic microparticle cavities. As discussed in the main manuscript, the surface wettability of the discretely patterned hydrophilic patches had a force balance with the water-oil interfacial tension, resulting in a different number of droplets clinging to the inner layer of the particle. As H_d/H_c (V_d/V_c) changed from 0.75 to 0.35, a single droplet gradually transitioned to several individual droplets within the square, circular, pentagonal, and hexagonal amphiphilic microparticles. The triangular shape behaved exceptionally differently, which would be discussed later. For H_d/H_c = 0.25, we obtained four, five, and six segmented droplets, respectively, formed at the discrete hydrophilic corners of the square, pentagonal, and hexagonal-shaped particles. For the circular particle with four hydrophilic patches, we obtained four isolated droplets only when H_d/H_c = 0.15 (data not shown in Figure S3). In the triangular amphiphilic microparticle, the hydrophilic patches were relatively spaced farther apart compared to the other shapes, which resulted in an immediate splitting of the three droplets at the hydrophilic corners across all H_d/H_c ratios. We have computed a ratio, R_c/R_{PEG}, of the radius of the inscribing circle passing through the centers of hydrophilic patches (R_c) to patch radius (R_{PEG}) to describe the anomalous trend for triangular particles. We obtained R_c/R_{PEG} = 5.56 for the triangular particles, which was clearly higher than the other particle shapes with R_c/R_{PEG} < 4. We have further computed a ratio, d_{PEG}/R_c, where d_{PEG} is the distance between adjacent hydrophilic patches. Even though these microparticles had different d_{PEG}/R_c ratios, the droplet transition trend within them was similar (apart from the triangular particles).

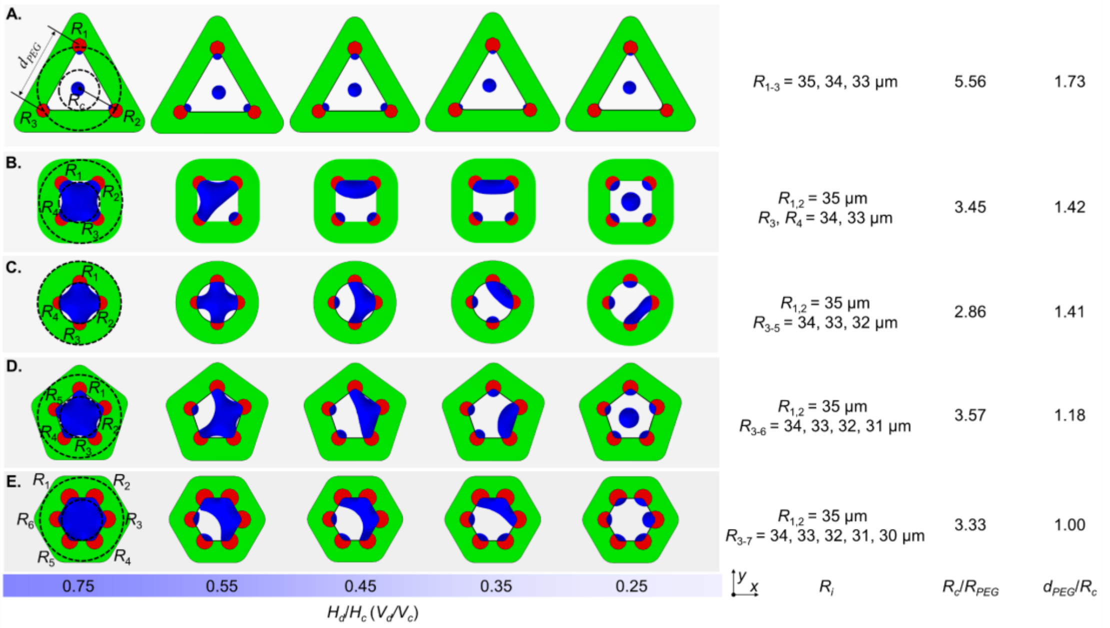

Figure S3. Numerical dropicle formation within triangular, square, circular, pentagonal, and hexagonal-shaped amphiphilic microparticles with evenly patterned hydrophilic patches. The circular, pentagonal, and hexagonal particles enable a similar transition trend with the 4C dropicles, from a single droplet to multiple small droplets at the corners, for decreasing H_d/H_c (V_d/V_c) ratio. For the triangular particle, three droplets are formed at the corners across all H_d/H_c ratios. Two dotted circles represent the inscribed circles tangent to the inner and outer boundaries of the particles, respectively.

To further clarify this anomalous behavior of the triangular-shaped particles, we resized our particles to have a constant radius of the inscribed circle (R_c) passing through all the hydrophilic patches irrespective of the particle shape (**Figure S**4). The dropicle transition trend was similar across all these amphiphilic microparticles regardless of shape, transitioning from a whole droplet to multiple small droplets at the corners, for decreasing H_d/H_c (V_d/V_c) ratios. A constant ratio of R_c/R_{PEG} = 3.45 for all the particle shapes, where the patch radius (R_{PEG} = R_1 = 35μm) to the inscribed circle radius (R_c = 120μm) that passed through the patch centers, was important to enable a similar trend from single to multiple droplets. The particle shape did not impact the dropicle formation trend when properly normalized. Notably, the d_{PEG}/R_c value of 1.77 for the triangular particles in Figure S4A was not very different from 1.73 in Figure S3A. This confirmed that the ratio d_{PEG}/R_c was not important in determining the dropicle configuration. It was the relative size of the hydrophilic patches R_{PEG} with respect to the radius R_c that controls the dropicle configuration.

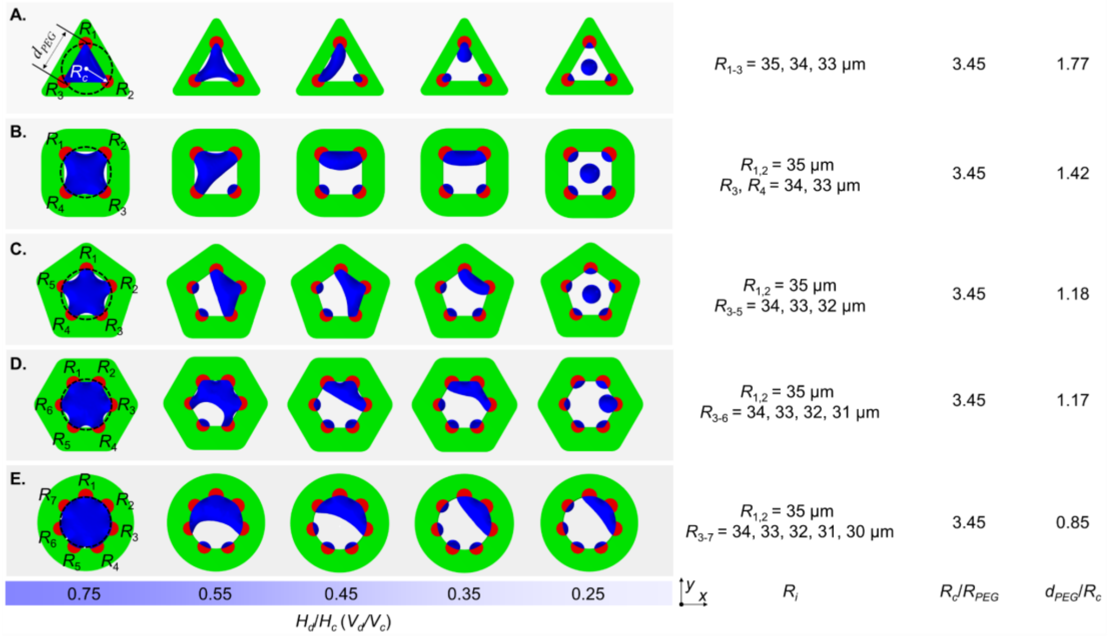

Figure S4. Numerical dropicle formation within triangular, square, pentagonal, hexagonal, and circular-shaped amphiphilic microparticles with three, four, five, six, and seven evenly patterned hydrophilic patches. A constant ratio R_c/R_{PEG} = 3.45 ensures that all the triangular, square, pentagonal, hexagonal, and circular particles enable a similar transition trend, from a whole droplet to multiple small droplets at the corners, for decreasing H_d/H_c (V_d/V_c) values.

Additional experimental results on dropicle formation:

We plotted the experimental average volumes and their standard deviation of all individual S^1, T^{+1d}, R^{+2d}, and C^{4d} ($[C^{4d}]_I$ and $[C^{4d}]_{II}$) droplets within F_1, F_9, F_{12}, and F_{16} particles (**Figure S5**). For a small proportion of the $[C^{4d}]_I$ dropicles, the average volume of the large droplet was even higher than the R^{+2d} droplet, which may result from the difference in droplet shrinkage within each 4C particle.

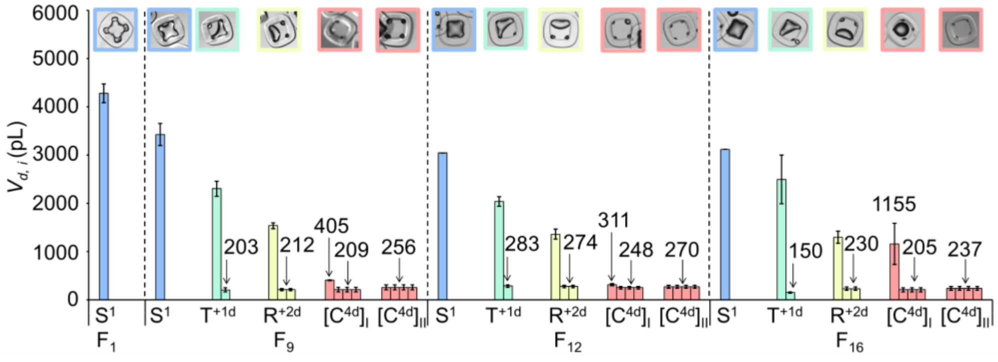

Figure S5. Experimental volume distributions of individual droplets within F_1, F_9, F_{12}, and F_{16} particles.

Figure S6A depicts the F_2 and F_4 dropicle formation, where the particles in EtOH and PBS, and the dropicles in oil (bright field and fluorescent) can be seen. We obtained S^1 (~40%) and T^{+1d} (~60%) dropicles within both particles (Figure S6B).

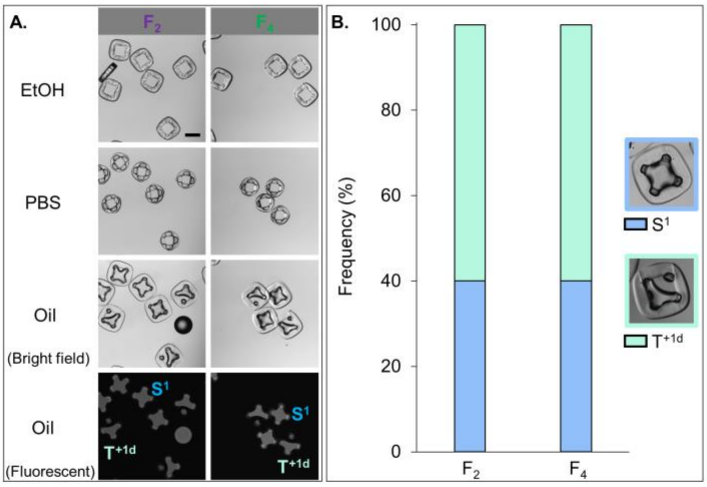

Figure S6. F_2 and F_4 dropicle formation. Scale bar: 200µm.

Numerical simulations of dropicle formation using adaptive meshing:

We used "Adaptive Meshing" for modeling the dropicle formation inside a 4C amphiphilic particle (**Figure S7**). We generated a "physics-controlled mesh" with an "extremely coarse" element size (Mesh 1). We generated additional Meshes 2-11 based on Mesh 1 using "Adaptive Meshing". Here, we set an "error estimation parameter" under Study → Solver Configurations → Solution 1 (sol1) → Time Dependent Solver → Adaptive Mesh Refinement as: "sqrt(comp1.phipfx^2 + comp1.phipfy^2 + comp1.phipfz^2)", where comp1.phipfx, comp1.phipfy, and comp1.phypfz denoted the x, y, and z components of the phase field variable (Φ_{pf}). This "error estimation parameter" refined the moving mesh along the water-oil interface. A zoomed-in Mesh 3 showed the adaptive mesh within the particle cavity. A refined mesh followed the water-oil interface with varying T^*. In this particular example shown in Figure S7, four individual droplets were formed at the corners of the particle and one at the center eventually, following the dropicle transition trend of $S^{1} \rightarrow T^{+1d} \rightarrow R^{+2d} \rightarrow C^{4d}$. One can clearly follow the mesh refinement along the partitioned droplets at the four corners and at the center of the cavity.

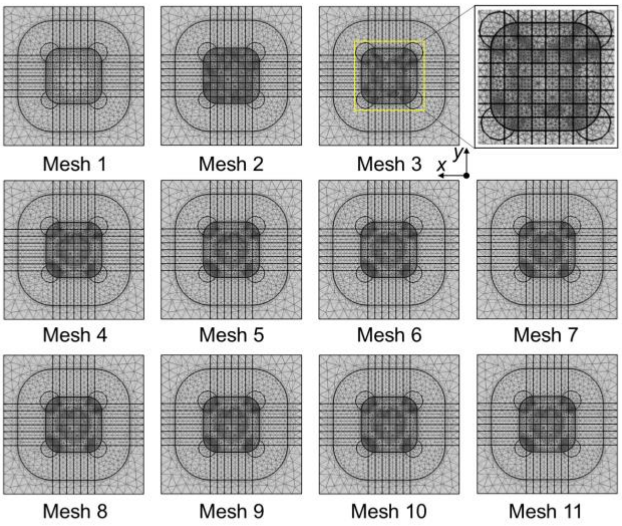

Figure S7. Adaptive meshes varying with T^* (0-1) for the dropicle formation with H_d/H_c = 0.25, and $R_{1,2}$, R_3, R_4 = 35µm, 33µm, 32 µm.

Experiment setup for the particle fabrication:

Figure S8 showed the experiment setup for the 4C amphiphilic microparticle fabrication with the stop-flow lithography.

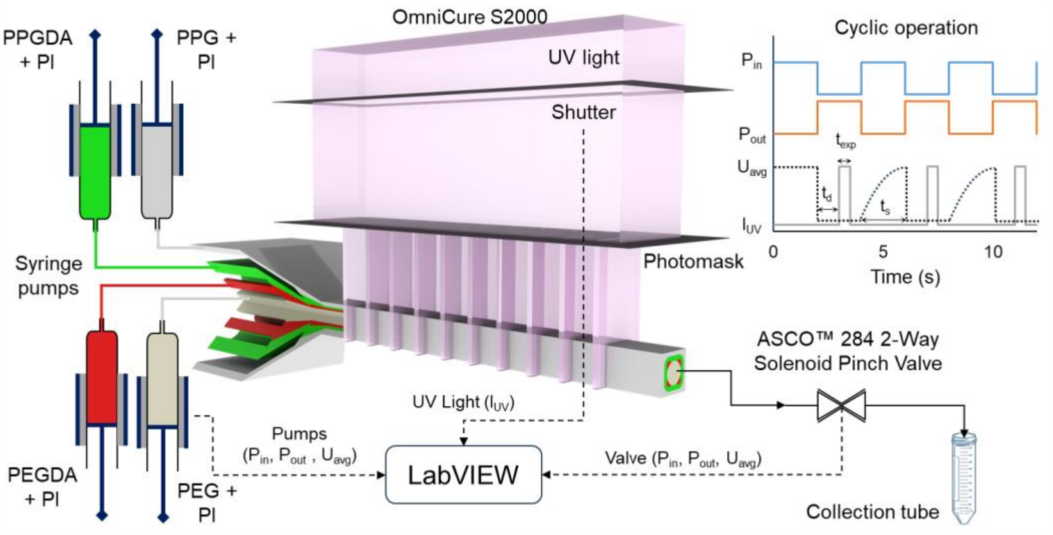

Figure S8. Experiment setup for fabricating 4C amphiphilic microparticles. Using the stop-flow lithography technique, the four polymer precursors (PPG, PPGDA, PEGDA, and PEG) mixed with a photo-initiator (PI) are sculpted through the microfluidic device, and afterward, the co-flow streams are cured under the photomask that shapes the UV light to 100 µm thickness. The cured 4C amphiphilic microparticles are collected in a tube.

Modeling hydrophilic patches to capture the experimental variabilities:

Considering the inhomogeneity in the fabricated particle dimensions, we intentionally differentiated $R_{1,2}$, R_3, and R_4 in the numerical simulations to break the symmetry of the 4C particles and include the variability in the particle dimensions. Thus, we gave a relatively reasonable radius difference of ~5-10% among the four hydrophilic patches per particle by setting the patch radii $R_{1,2}$, R_3, and R_4 as 50, 47, and 45 µm (large), 35, 33, and 32 µm (medium), and 20, 19, and 18 µm (small), where the four patch radii varied by 1-5µm within a given particle type. The average patch radii for the large, medium, and small-patch particles had a difference of at least 10 µm.

The experimentally measured average patch diameter D_{PEG} was similar to the numerical values (Figures 2F and 3B). For a clear comparison of the hydrophilic patch radii between simulation and experiment, we added **Table S1** to show the experimental average values (~24-48 µm) and standard deviations (~2.1-2.7 µm) of the patch radius $R_{PEG} = D_{PEG}/2$ for F_i particles in EtOH, where i = 1, 2, 4, 6, 9, 12, and 16, respectively. The experimental coefficient of variation (CV = SD/R_{PEG}) among the hydrophilic patches for each particle was derived as ~6%-10% in EtOH, aligning well with the numerical values. Besides, the experimental average patch radii for F_1,

F_9, and F_{12} particles had a difference of ~10 μm, similar to the numerical data of the large, medium, and small-patch particles in the simulations, respectively.

Table S1. Average values of R_{PEG} ($D_{PEG}/2$), their standard deviations (*SD*), and coefficient of variation (CV) of F_i particles in diverse media.

F_i	Medium	D_{PEG}	R_{PEG}	*SD*	CV (%)	Numerical $R_{1,2}$,R_3,R_4
	EtOH	95	48	2.7	5.7	
F_1	PBS	118	59	2.4	4	
	Oil	97	48	2.8	5.7	
	EtOH	91	45	2.6	5.8	50,47,45 μm
F_2	PBS	107	53	2.5	4.7	(Large)
	Oil	83	41	2.7	6.5	
	EtOH	83	42	2.6	6.2	
F_4	PBS	99	49	2.6	5.2	
	Oil	67	33	2.7	8	
	EtOH	71	36	2.1	5.8	
F_9	PBS	81	40	2.3	5.7	35,33,32 μm (Medium)
	Oil	52	26	2.3	8.8	
	EtOH	52	26	2.2	8.4	
F_{12}	PBS	62	31	2.2	6.9	
	Oil	36	18	2.2	12.4	20,19,18 μm
	EtOH	47	24	2.3	9.7	(Small)
F_{16}	PBS	56	28	2.5	8.8	
	Oil	32	16	2.3	14.4	

Movies captions:

Movie S1. Numerical simulations of S^1, T^{+1d}, R^{+2d} and C^{4d} dropicles formed within a 4C particle with large, medium, medium, and small size hydrophilic patches, and H_d/H_c of 0.75, 0.55, 0.35, and 0.25, respectively.

Movie S2. Experimental dropicle formation within F_9 particles captured for ~45min. The dropicle configuration changed from S^1 to T^{+1d}.

Movie S3. Experimental dropicle formation within F_{16} particles captured for ~3h. The dropicle configuration transitioned from S^1 to T^{+1d} to R^{+2d} to C^{4d}.

COMSOL models:

Model S1. 'Two-Phase Flow, Level Set' model for predicting the shape of the cross-sectional flow profile associated with F_1 particles.

Model S2. 'Two-Phase Flow, Phase Field' model for obtaining various dropicle configurations with H_w/H_c = 0.75, and $R_{1\text{-}4}$ = [50, 50, 47, 45 μm].

Device model for 3D printing:

Model S3. A computer-aided design (CAD) model in the stereolithography (STL) file format is provided for 3D printing the device with multiple integrated microfluidic channels.
